# Verification of Shoulder External Rotators Strength Measurement Using a Suspension Scale

**DOI:** 10.7759/cureus.26106

**Published:** 2022-06-20

**Authors:** Atsushi Ueda, Yasuhiro Mitani, Hitoshi Koda, Toshimitsu Omine, Ryuta Inada, Naoyuki Konishi, Shunsai Mori

**Affiliations:** 1 Department of Rehabilitation, Hankai Hospital, Sakai, JPN; 2 Department of Rehabilitation Sciences, Faculty of Allied Health Sciences, Kansai University of Welfare Sciences, Kashiwara, JPN; 3 Department of Rehabilitation, Shimada Hospital, Habikino, JPN; 4 Department of Physical Therapy, Osaka Isen, Osaka, JPN; 5 Department of Rehabilitation, Miyama Clinic, Amagasaki, JPN

**Keywords:** portable device, reliability, measurement, strength, shoulder

## Abstract

Background

Of the shoulder external rotators, the infraspinatus and teres minor are the key muscles that contribute to the dynamic stability of the shoulder. It is crucial to properly measure the strength values to evaluate muscle function and training load for shoulder external rotators. A suspension scale (SPS) can measure the mass of the suspended object, and it may be possible to apply it to measure strength. However, the utility of strength measurements using an SPS has not been clarified in previous studies. In this study, we aimed to investigate the intra-rater reliability of measuring the strength of shoulder external rotators using an SPS and the relationship between strength measurement using an SPS and a handheld dynamometer (HHD).

Methodology

The participants were 10 healthy males with 20 shoulders (24.5 ± 2.5 years old; height = 172.8 ± 5.4 cm; weight = 69.6 ± 8.1 kg). Upper extremity strength was measured at 90° shoulder abduction, 90° external rotation, 0° horizontal adduction/abduction, 90° elbow flexion, and 0° forearm pronation/supination in the prone position. The isometric strength of shoulder external rotation was measured with the SPS and HHD, and one examiner measured the maximum strength value. The intra-rater reliability of the two methods using SPS and HHD was evaluated using the intraclass correlation coefficient (ICC_1,2_), standard error of measurement (SEM), minimum detectable change (MDC), and Bland-Altman analysis. The relationship between the SPS and HHD was calculated as the correlation coefficient between the strength values of SPS and HHD.

Results

The intra-rater reliability of the strength measurement of shoulder external rotators using SPS was ICC_1,2_ 0.98 (95% confidence interval = 0.95-0.99), and SEM and MDC were 0.3 and 0.9, respectively. The measurements using SPS had no fixed and proportional biases. A significant positive correlation was observed between SPS and HHD (r = 0.94, p < 0.01).

Conclusions

The SPS is an alternative to the HHD for measuring the strength of shoulder external rotators. Thus, measuring the strength of shoulder external rotators using an SPS may be applied as a cost-effective and portable assessment method for shoulder function.

## Introduction

Although the glenohumeral joint (GHJ) has a large range of motion owing to the small size of the glenoid fossa with the humeral head, the joint structure has minimal bony stability [1,2]. To compensate for GHJ instability, the rotator cuff, composed of the infraspinatus, teres minor, supraspinatus, and subscapularis muscles, contributes to dynamic stabilization [3]. Particularly, the infraspinatus and teres minor muscles contribute to suppressing the anteroposterior and superior translation of the humeral head during shoulder elevation motion [3]. In contrast, weakness of shoulder external rotators such as the infraspinatus and teres minor muscles increases humeral head translations during shoulder motion [4]. Hence, shoulder external rotator weakness is involved in the onset of shoulder joint disorders, such as rotator cuff injuries [5,6], labral injuries [5,7], and subacromial impingement [8]. Moreover, Clarsen et al. reported that overhead handball athletes with shoulder external rotator weakness had an increased risk of shoulder disorders [9]. Thus, it is crucial to properly assess the strength of the shoulder external rotators for physical therapy and injury prevention.

The isokinetic dynamometer allows us to measure the strength of the shoulder external rotators throughout the range of motion at various motion speeds and testing modes (i.e., concentric, isometric, and eccentric modes) [10]. Previous studies have reported that an isokinetic dynamometer has excellent reliability in measuring strength [11]. However, they are expensive and not portable [12]. The handheld dynamometer (HHD) is a portable device that is used as a standard method to assess maximum strength without limiting the measurement surroundings [12]. Hence, it is critical to develop simple methods of strength measurement that can be used in a variety of fields with various portable devices such as the HHD.

Thus, we developed a simple method to measure the strength of the shoulder external rotators using a suspension scale (SPS). An SPS can weigh the mass of a suspended object. Moreover, they are low-cost and portable. Therefore, measurement of shoulder external rotator strength using an SPS may represent an inexpensive means to provide accurate measurements of the maximum strength. Hence, an SPS may be used to measure the strength of the shoulder external rotators. However, in previous studies, the utility of strength measurements using an SPS has not been clarified. Thus, we hypothesized that the SPS could be used as a reliable method for measuring the isometric strength of shoulder external rotators. Moreover, strength measurements of the shoulder external rotators using the SPS correlate with those using the HHD. The primary aim was to establish the intra-rater reliability of measuring the strength of shoulder external rotators using the SPS. The secondary aim was to investigate the relationship between the strength values acquired using the SPS and HHD.

## Materials and methods

Research design

This was a cross-sectional reliability study.

Participants

In total, 10 healthy men with 20 shoulders (age = 24.5 ± 2.5 years; height = 172.8 ± 5.4 cm; weight = 69.6 ± 8.1 kg) were included in this study. Patients with shoulder pain and an upper extremity surgical history were excluded from this study. This study was conducted in accordance with the Declaration of Helsinki and was approved by the ethical review board of Hankai Hospital (approval number: 2022-001). Written informed consent was obtained from all participants.

Methods

**Figure 1:**
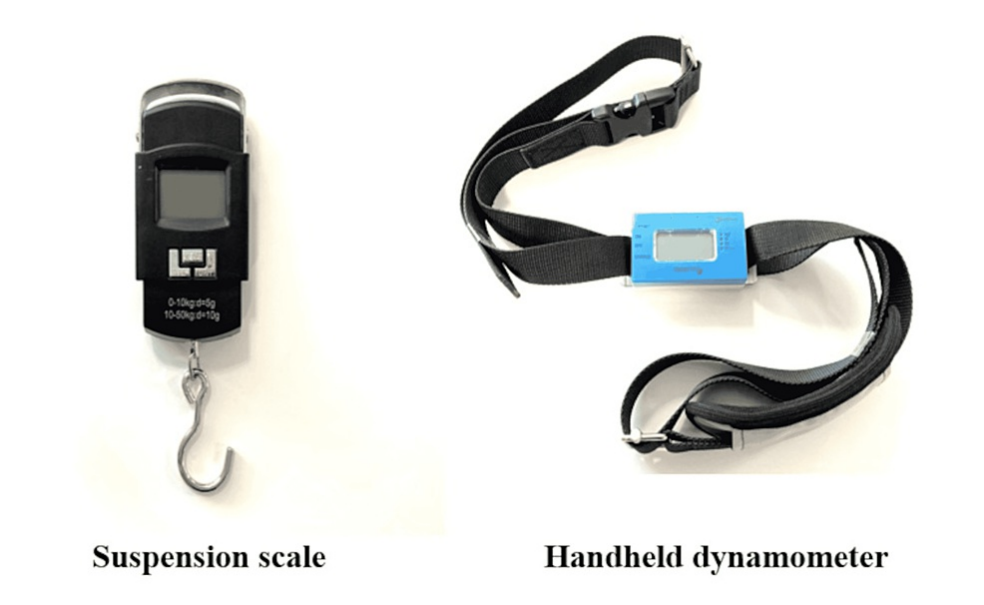
Suspension scale and handheld dynamometer.

To measure the strength of the shoulder external rotators, the SPS (Guangzhou Weiheng Electronics Co., Ltd., Guangdong, China) and pull-type HHD (Sakai Medical Co., Ltd., Tokyo, Japan) (Figure 1) were used. The SPS and pull-type HHD can measure the strength of pulling non-elastic belts attached to both ends of each device. The SPS and HHD can measure from 0.00 kg to 50.00 kg and from 0.0 kg to 150.0 kg, respectively.

**Figure 2:**
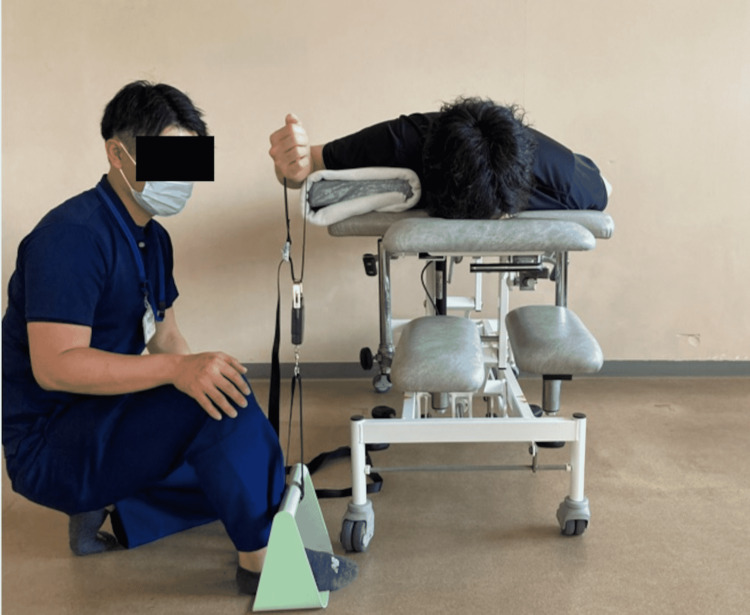
Strength measurement of shoulder external rotators using a suspension scale.

Regarding the experimental setting, to measure the strength of the shoulder external rotators, the participants were laid in a prone position on the bed in the following positions: 90° shoulder abduction, 90° external rotation, 0° horizontal adduction/abduction, 90° elbow flexion, and 0° forearm pronation/supination (Figure 2). Additionally, to maintain the measurement position of 0° shoulder adduction/abduction, the proximal humerus was placed on a 5 cm platform.

**Figure 3:**
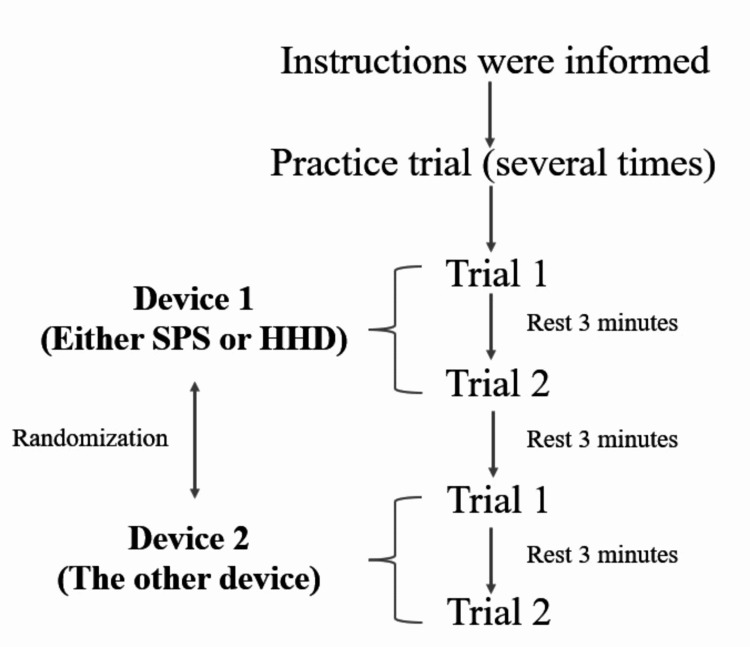
Experimental protocol of strength measurement. SPS: suspension scale; HHD: handheld dynamometer

The experimental protocol of this study is shown in Figure 3. One non-elastic belt attached to both ends of the SPS was vertically fixed on the distal forearm, and the other was immobilized on the bar fixed on the floor (with loading lower extremity of one examiner) (Figure 2). The strength measurement of the HHD was performed using the same method (experiment setting). Both devices were removed and the experimental setup was returned to the same condition after each test. Prior to the measurement, the measurement method instructions (SPS and HHD) were explained to the participants, and each strength measurement was practiced several times. The isometric strength of the shoulder external rotators was measured for five seconds to achieve maximum strength [13]. The measurements were taken twice by a physical therapist with more than nine years of experience in the rehabilitation of musculoskeletal disorders. Rest time after strength measurement was a three-minute break between the strength measurement of each device (SPS and HHD) and trials [11,14]. The order of the tests was determined by randomization to minimize the effects of fatigue with repeated measurements.

Statistical analysis

To evaluate the intra-rater reliability of the strength measurement for the shoulder external rotators using the SPS and the relationship between the strength values of the SPS and HHD, a statistical analysis was conducted using the following procedure. First, to confirm the normality of each measurement data, the Shapiro-Wilk test was performed, and normality was defined as a p-value of ≥0.05. Second, the intraclass correlation coefficient (ICC_1,2_), standard error of measurement (SEM), and 95% confidence interval (95% CI) were calculated to evaluate the intra-rater reliability of the two measurements using SPS and HHD. SEM was calculated as SD×$\sqrt{1-ICC}$. The reliability was evaluated as excellent reliability (>0.90), good reliability (0.75-0.90), moderate reliability (0.50-0.75), and poor reliability (<0.50) [15]. The minimum detectable change (MDC) of the SPS and HHD measurements was calculated as SEM×1.96×$\sqrt{2}$ [12]. Third, an evaluation of absolute reliability with Bland-Altman analysis was performed to assess the presence of fixed and proportional biases. Fixed bias was calculated as the 95% CI of the mean difference between the two trials for each measurement. Additionally, a fixed bias was considered positive if 0 was not included within the 95% CI of the SPS and HHD measurements. To determine the proportional bias, we calculated the regression equation based on the difference in values and mean values of the two methods. If the regression was significant, the proportional bias was positive. Finally, the correlation coefficient between the strength values of the SPS and HHD was calculated to investigate the utility of the strength measurement of the shoulder external rotators using SPS. If normality data was confirmed, one correlation coefficient was calculated using Pearson’s correlation coefficient. Otherwise, it was calculated using Spearman’s rank correlation coefficient. Statistical analyses were performed using SPSS Statistics version 27 (IBM Corp., Armonk, NY, USA). The significance level was set at 5% (p < 0.05).

## Results

The measured values of SPS and HHD and the results of ICC_1,2_, SEM, and MDC are shown in Table 1. Measured values of the SPS and HHD were 5.8 ± 2.3 kg and 6.1 ± 2.4 kg (SPS) and 5.6 ± 2.4 kg and 5.9 ± 2.4 kg (HHD) for the first and second trials, respectively. The Shapiro-Wilk test for all measurement data confirmed the normality of all data. The ICC_1,2_ (95% CI) for the SPS and HHD were 0.98 (0.95-0.99) and 0.92 (0.80-0.97), respectively. The SEM were 0.3 and 0.6, respectively. The MDC were 0.9 and 1.7, respectively.

**Table 1:** Strength values and intra-rater reliability, standard error of measurement, and minimal detectable change of suspension scale and handheld dynamometer. ^a^Mean ± standard deviation. ICC: intraclass correlation coefficient; 95% CI: 95% confidence interval; SEM: standard error of measurement

Measurement device	Strength values^a^						Intra-rater reliability		Minimal detectable change
	Trial 1			Trial 2			ICC [95% CI]	SEM	
Suspension scale (kg)	5.8	±	2.3	6.1	±	2.4	0.98 [0.95–0.99]	0.3	0.9
Handheld dynamometer (kg)	5.6	±	2.4	5.9	±	2.4	0.92 [0.80–0.97]	0.6	1.7

The results of the Bland-Altman analysis for the SPS and HHD measurements are shown in Table 2. The Bland-Altman analysis showed that there were no fixed and proportional biases in the measurements of SPS and HHD.

**Table 2:** Bland-Altman analysis of suspension scale and handheld dynamometer. ^a^Values less than 0.05 were considered statistically significant. 95% CI: 95% confidence interval

Measurement device	Fixed bias		Proportional bias		
	95% CI	Results	Slope of regression line	P-value^a^	Results
Suspension scale	-0.52–0.03	No	0.01	0.86	No
Handheld dynamometer	-0.90–0.25	No	-0.18	0.17	No

**Figure 4:**
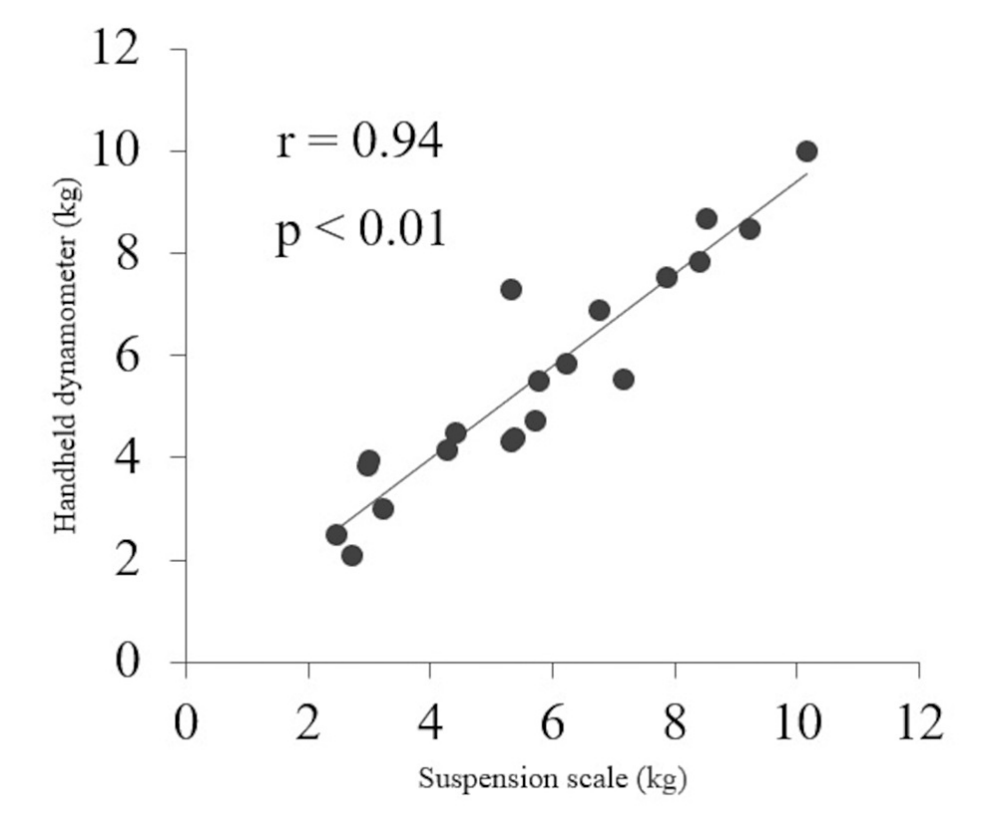
Scatter plot of strength values (average of two measurements) of the suspension scale and handheld dynamometer.

A scatter plot of the mean values of the SPS and HHD data is shown in Figure 4. Correlation analysis showed a significant positive correlation between the strength values of SPS and HHD (r = 0.94, p < 0.01).

## Discussion

This study aimed to clarify the intra-rater reliability of measuring the strength of shoulder external rotators using an SPS and the relationship between the strength measurement using the SPS and HHD. Our study found that the intra-rater reliability of measuring the strength using the SPS and HHD were ICC 0.98 and 0.92, respectively. In addition, both devices had excellent reliability. The SEM and MDC of the SPS were smaller than those of the HHD. The results of the Bland-Altman analysis showed that there were no fixed and proportional biases in measurements using the SPS and HHD. The correlation analysis showed that there was a strong correlation (positive) between the strength values of the SPS and HHD (r = 0.94, p < 0.01). Therefore, the study hypothesis was supported by the results.

Regarding the reliability of measuring strength for shoulder external rotators, Papotto et al. reported that using an isokinetic dynamometer had an ICC of 0.92 [11]. Additionally, Cools et al. and Furness et al. demonstrated that the reliability of a push-type HHD was ICC 0.93 and 0.98, respectively [13,16]. A push-type HHD is a measurement equipment that can assess strength by applying pressure to the sensor. The reliability of the strength measurement for the shoulder external rotators using the SPS and HHD was ICC 0.98 and 0.92, respectively. Hence, the measurement methods used in our study were similar to those used in previous studies.

Our study revealed the intra-rater reliability of measuring the strength of shoulder external rotators by using an inexpensive SPS. Moreover, the results showed that the strength measurements of the shoulder external rotators using the SPS strongly correlated with those using the HHD. Regarding the clinical aspects of shoulder external rotators, decreased strength and muscle activity of the infraspinatus and teres minor muscles in shoulder external rotation were observed in patients with various shoulder disorders such as subacromial impingement [17], rotator cuff injuries [18], and shoulder instability [19]. Thus, proper assessment of the strength of shoulder external rotators is fundamental in clinical practice [20]. Our study indicated that strength measurement of shoulder external rotators using a portable SPS was cost-effective and an alternative to the HHD. Hence, the measurement method in our study can be used not only in the clinical practice of physical therapy but also in a wide range of fields, such as medical examinations, exercise, and training instruction for athletes and elderly people in the community.

Limitations of the study

Our study has a few limitations. First, we could not clarify the internal validity of the strength measurement of shoulder external rotators using the SPS, although it strongly correlated with that of the HHD. However, to investigate the internal validity of the measuring strength method with a portable device such as the HHD, previous studies have used a strength value acquired with an isokinetic dynamometer as the gold standard [21,22]. We could not determine the relationship between strength values using the SPS and isokinetic dynamometer. Second, we could not perform measurements with the SPS and HHD in parallel. Hence, the concurrent reliability and validity using the SPS were unclear. However, simultaneous measurement using the SPS and HHD using one belt may be inaccurate due to a bias of the load on a belt. Therefore, in this study, the measurements using the HHD and SPS were separately performed in accordance with the previous study [21]. Third, we could not investigate the inter-rater reliability of the strength measurement using the SPS. Fourth, the study participants were healthy males, and it is unclear whether in this study the measurements using the SPS could be applied to subjects with other backgrounds, such as women and elderly people. Therefore, further studies including females and a wider age group are necessary.

## Conclusions

This study aimed to investigate the utility of strength measurement of shoulder external rotators using an inexpensive and portable SPS. Results showed excellent reliability. Additionally, the strength value obtained using the SPS showed a strong correlation with that of the HHD. In conclusion, these results indicate that the SPS can be used as a clinically useful, cost-effective, and portable assessment method for shoulder function.
